# Conductive and Adhesive Granular Alginate Hydrogels for On-Tissue Writable Bioelectronics

**DOI:** 10.3390/gels9020167

**Published:** 2023-02-19

**Authors:** Sumin Kim, Heewon Choi, Donghee Son, Mikyung Shin

**Affiliations:** 1 Department of Intelligent Precision Healthcare Convergence, Sungkyunkwan University (SKKU), Suwon 16419, Republic of Korea; 2 Department of Electrical and Computer Engineering, Sungkyunkwan University (SKKU), Suwon 16419, Republic of Korea; 3 Department of Superintelligence Engineering, Sungkyunkwan University (SKKU), Suwon 16419, Republic of Korea; 4 Department of Biomedical Engineering, Sungkyunkwan University (SKKU), Suwon 16419, Republic of Korea

**Keywords:** alginate, PEDOT:PSS, granular hydrogel, injectable hydrogel, conductive hydrogel, tissue-adhesive hydrogel, on-tissue writable, bioelectronics

## Abstract

Conductive hydrogels are promising materials in bioelectronics that ensure a tissue-like soft modulus and re-enact the electrophysiological function of damaged tissues. However, recent approaches to fabricating conductive hydrogels have proved difficult: fixing of the conductive hydrogels on the target tissues hydrogels requires the aids from other medical glues because of their weak tissue-adhesiveness. In this study, an intrinsically conductive and tissue-adhesive granular hydrogel consisting of a PEDOT:PSS conducting polymer and an adhesive catechol-conjugated alginate polymer was fabricated via an electrohydrodynamic spraying method. Because alginate-based polymers can be crosslinked by calcium ions, alginate-catechol polymers mixed with PEDOT:PSS granular hydrogels (ACP) were easily fabricated. The fabricated ACP exhibited not only adhesive and shear-thinning properties but also conductivity similar to that of muscle tissue. Additionally, the granular structure makes the hydrogel injectable through a syringe, enabling on-tissue printing. This multifunctional granular hydrogel can be applied to soft and flexible electronics to connect humans and machines.

## 1. Introduction

Recently, interest in conductive hydrogels has increased in a wide range of fields from biomedical engineering to flexible electronics [1,2,3]. In addition to their conductivity, the soft modulus matching that of human tissue makes conductive hydrogels promising candidates for bridging electronics and human beings [4,5,6,7]. Conductive hydrogels have been used in various fields, such as electroactive tissue engineering (e.g., cell scaffolds) [8,9] and bioelectronics (e.g., wearable or implantable electronics) [10,11,12]. One of the form factors of conductive hydrogels is a patch, which can be attached or affixed to record electrical signals on skin tissues or enhance tissue conduction [13,14,15]. However, the patch-type hydrogels can exhibit relatively low moisture/gas/liquid permeability and induce acute inflammation or irritation when applied to the target region for a long-term period [16,17]. Moreover, if they are used as substrates for implantable electronics, an invasive surgical procedure is required for applying the patch to the tissue interfaces. To resolve these limitations, injectable and conductive hydrogels can be one of the candidate materials for advanced wearable/implantable bioelectronics [18,19,20].

To achieve great injectability and enhanced gas permeability of the hydrogels, the granular structure can be considered. According to previous reports, such granular hydrogels can promote cell invasion due to their intrinsic microporous structures, leading to rapid tissue regeneration [17,20]. The injectable hydrogels can be applied to the target tissue in a minimally invasive way [17,20]. A few studies have focused on such granular structures for the fabrication of conductive hydrogels [20]. Among them, several studies on developing conductive and injectable hydrogels have suggested using metal-phenolic networks for continuous electrical flow [19,20]. In those hydrogels, phenolic groups can also show tissue adhesiveness inspired by marine mussels’ adhesion mechanism. However, there still remain challenges for retaining stable tissue adhesiveness, high conductivity and injectability of the hydrogels. For instance, while the metal ions/nanoparticles (e.g., Ag, Fe ions) can enhance the conductivity, the metals play a role as oxidants for the phenolic groups, and the oxidized phenolic groups exhibit unstable adhesiveness and injectability. Furthermore, the mechanical modulus of the hydrogels gradually increases due to additional phenolic crosslinking, which can lead to modulus mismatching between hydrogels and tissues as time goes by [19,20,21]. Therefore, to maintain the adhesiveness of the phenolic groups in conductive and injectable hydrogels, another conductive additive, such as a conductive polymer, poly(3,4-ethylenedioxythiophene):poly(styrenesulfonate) (PEDOT:PSS), not metals, can be considered as a component for these hydrogels. In particular, PEDOT:PSS is a widely used wet-conducting polymer with high conductivity and biocompatibility required for bioelectronics [8,22,23,24].

**Figure 1:**
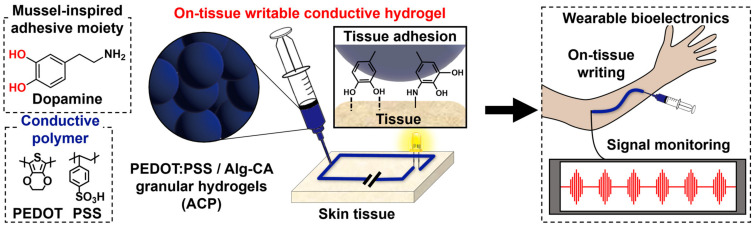
Overall schematic of the on-tissue writable conductive hydrogel.

Herein, we report a new type of conductive and adhesive granular hydrogel that can be directly written onto the tissue (Figure 1). Using alginate-based tissue-adhesive polymers with mussel-inspired catechol moieties (e.g., catechol-conjugated alginate; Alg-CA), we fabricated a tissue-adhesive granular hydrogel (e.g., a microgel) via an electrohydrodynamic spraying strategy, which is a facile and time-saving technique compared with that of a microfluidic device [25]. To improve its electrical conductivity, we introduced PEDOT:PSS into the microgel. The granular structure can enhance the surface area that each PEDOT:PSS/Alg-CA microgel contacts, allowing great conductivity of the hydrogels by the continuous electrical flow. The fabricated PEDOT:PSS/Alg-CA granular hydrogel (ACP) also exhibits tissue-adhesive properties via hydrogen bonding or covalent bonding between the functional groups of the tissue surface and catechol moieties of hydrogels [26,27,28]. Such adhesiveness between the ACP hydrogels and tissue interfaces enables on-skin printing of the hydrogels. Thus, the enhanced conductivity and intrinsic injectability of the ACP make it a promising material for flexible bioelectronics [29,30,31]. Ultimately, when compared with conventional bulk hydrogels, such conductive and adhesive granular hydrogels can be potential candidate materials for soft electrodes with great printability/injectability, high gas permeability, and biocompatibility for future wearable on-skin bioelectronics.

## 2. Results and Discussion

### 2.1. Fabrication of Catechol-Conjugated Alginate Granular Hydrogels (AC)

**Figure 2:**
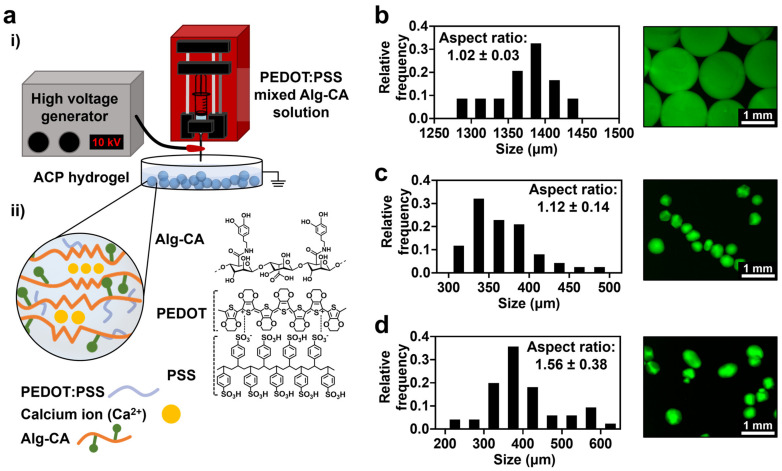
Experimental setup and fabrication of Alg-CA granular hydrogel. (**a**): (**i**) Illustration of the experimental setup; (**ii**) Chemical structure and illustration of the granular hydrogel. (**b**–**d**) Size distribution and fluorescence images of AC fabricated with different voltages; 5 kV (**b**), 10 kV (**c**), and 15 kV (**d**). Size and aspect ratio were measured from fluorescence images.

Oxidation is a common method to fabricate hydrogels using catechol conjugated polymers. However, the crosslinking of polymer chains via catechol oxidation reduces the tissue-adhesive properties of the catechol groups [32]. Therefore, we decided to crosslink the catechol-conjugated alginate (Alg-CA) via ionic crosslinking using calcium ions to form a typical egg-box structure. To provide conductive properties to the hydrogels, PEDOT:PSS was mixed with Alg-CA. Micro-size granular structures obtained by the electrohydrodynamic spraying strategy allowed for the injectability of the hydrogels (Figure 2a).

First, the conditions for fabricating the micro-sized hydrogels were optimized using Alg-CA. Catechol moieties were conjugated to alginate as previously reported (Figure S1) [33]. In the electrohydrodynamic spraying method, the morphology of the fabricated hydrogels varied with the voltage used in the fabrication system [34]. The fabricated granular hydrogel made from Alg-CA was named AC. At low voltage (5 kV), the fabricated AC was spherical. However, its average size was bigger than 1000 µm, resulting in low injectability (Figure 2b). In contrast, a higher voltage (15 kV) formed smaller hydrogels, while increasing their aspect ratio (Figure 2d). For the fabrication of small and sphere-shaped granular hydrogels, 10 kV was appropriate (Figure 2c).

### 2.2. Mechanical Characterization of AC and PEDOT:PSS Mixed Alg-CA Granular Hydrogels (ACP)

**Figure 3:**
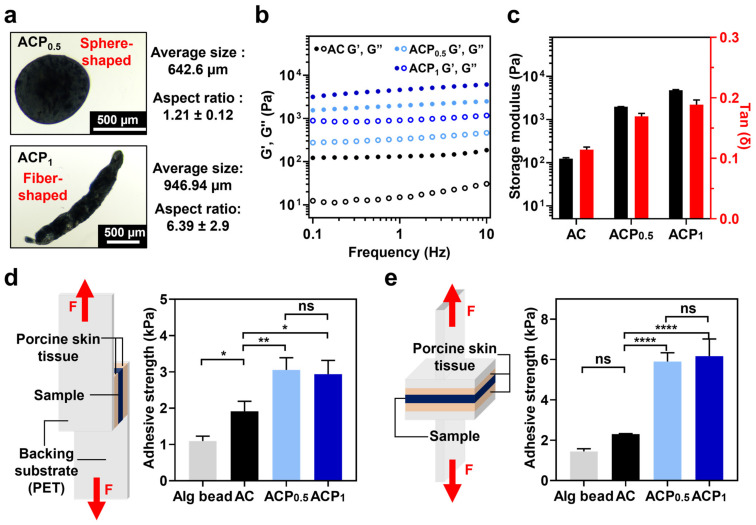
Mechanical characterization of AC and ACPs. (**a**) Morphology of fabricated ACPs. Optical images of ACP_0.5_ (top) and ACP_1_ (bottom) and their average size and aspect ratio. (**b**) Oscillation frequency sweep measurement of AC (black), ACP_0.5_ (light blue), and ACP_1_ (dark blue). Filled circles represent storage modulus (G′), and empty circles represent loss modulus (G″). (**c**) Storage modulus (black) and tan (δ) (red) value of each sample at the frequency of 1 Hz. (**d,e**) Adhesive strength of Alg bead, AC, ACP_0.5_, and ACP_1_ on porcine skin tissue with the application of shear stress (**d**) and tensile stress (**e**). Experimental setup for each test is demonstrated on the left side of each panel. One-way ANOVA; ns means not significant, * p < 0.05, ** *p* < 0.01, **** *p* < 0.0001.

The PEDOT:PSS solution was mixed with Alg-CA to form a new conductive granular hydrogel (ACP). As demonstrated, to form granular hydrogels, the voltage was fixed at 10 kV while the concentration of PEDOT:PSS varied (Table 1). The viscosity of the PEDOT:PSS solution increased compared with that of the pre-gel solution, resulting in morphological differences in the fabricated hydrogel at higher PEDOT:PSS concentrations. As shown in Figure 3a, ACP_1_ is a fiber-shaped hydrogel with a larger size and aspect ratio than ACP_0.5_, having a higher PEDOT:PSS concentration.

To estimate the mechanical durability while printing, the storage modulus (G′) and loss modulus (G″) of the fabricated hydrogels were measured while changing the frequency from 0.1 to 10 Hz with 1% shear strain (Figure 3b and Figure S2). All hydrogels maintained a tan(δ) value of less than 1, indicating retention of the hydrogel state (Figure 3c). ACP_1_ had a high storage modulus, followed by ACP_0.5_ and AC. Additionally, we confirmed the cross-sectional morphology of each hydrogel (Figure S3). The ACPs showed a much denser network and larger pores (14.9 ± 0.86 μm for ACP_0.5_ and 9.1 ± 1.39 μm for ACP_1_) than that of AC alone (24.5 ± 3.18 μm), which might be attributed to intermolecular interactions between Alg-CA and PEDOT:PSS, such as hydrophobic interactions. The increase in the modulus and high density of the hydrogels with the incorporation of more PEDOT:PSS would affect the physical stability of the materials in future applications.

### 2.3. Tissue Adhesive Property of AC and ACPs

To use the ACPs as on-skin printable hydrogels, the tissue adhesiveness of the ACPs was estimated using a universal testing machine (UTM). The adhesive strength was tested by applying shear (Figure 3d) and tensile stresses (Figure 3e). Alginate without any modifications was also fabricated as a granular hydrogel (Alg bead) and tested. Alg bead, AC, ACP_0.5_, and ACP_1_ samples were applied between porcine skin tissue and subjected to shear or tensile stress. The AC group exhibited higher adhesive strength than the Alg bead group. Under the tensile stress, the AC demonstrated increased adhesive strength (6 kPa), whereas under the shear stress, the adhesive strength of AC was lower (e.g., 3 kPa). This is due to their morphological characteristics of the granular hydrogels, which roll on the porcine skin surface in the direction of the shear force. The adhesive strength of the ACPs was also higher than that of the AC, regardless of the stress direction indicating that the adhesive strength is affected by the viscous PEDOT:PSS polymer, as well as by the catechol moieties. The results might be due to the prevention of cohesive failure in the hydrogel network via an intermolecular interaction between the Alg-CA backbone and the conductive polymer. To sum it up, the ACPs have strong tissue-adhesive properties and can be applied to on-skin printable materials.

### 2.4. Injectability of ACPs

**Figure 4:**
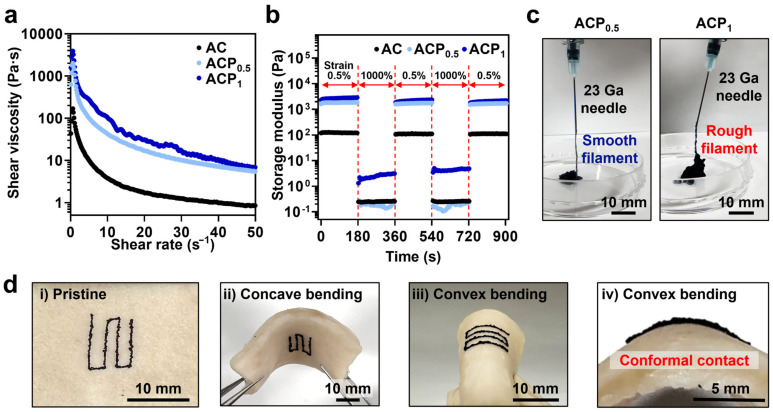
Injectability characterization of AC and ACP. (**a**) Shear-thinning behavior of AC and ACP. (**b**) Self-healing properties of AC (black), ACP_0.5_ (light blue), and ACP_1_ (blue). Storage modulus was plotted with filled circles. (**c**) Injection of ACPs using a 23 Ga needle. (**d**) Bending of ACP_0.5_ printed on the porcine skin tissue in the concave or convex directions.

The injectability of the ACPs was tested for future applications of ACPs as an on-tissue writable hydrogel. Based on the injectability of the granular hydrogels, their shear-thinning behavior was tested (Figure 4a). The ACPs showed higher shear viscosity than AC, because of the interactions (e.g., pi-pi stacking) between ring structures of Alg-CA and PEDOT:PSS. As the shear rate increased from 0 to 50 s^−1^, the shear viscosity decreased. The self-healing properties of ACPs, which originated from characteristics of the granular hydrogels, were also evaluated. The storage modulus at 0.5% strain recovered after a much higher strain (1000%) was applied (Figure 4b). The ACPs were injected using a 23 Ga needle with an inner diameter of ~340 µm (Figure 4c). The injected ACP_0.5_, which were spherical, formed smooth filaments. On the contrary, ACP_1_ with a fiber shape formed rough filaments. This makes ACP_0.5_ a promising candidate for highly injectable hydrogel systems. ACP_0.5_ was printed on porcine skin tissue to demonstrate its application as an on-tissue printable hydrogel. The hydrogel was printed in a complicated structure in a small area to demonstrate its highly injectable property. As shown in Figure 4d, ACP conformally attached to the skin tissue while bending in the concave or convex directions. This demonstrates that ACPs can be adapted to the motion of the skin while maintaining conformal contact with the tissue.

### 2.5. Electrical Conductivity of AC and ACPs

**Figure 5:**
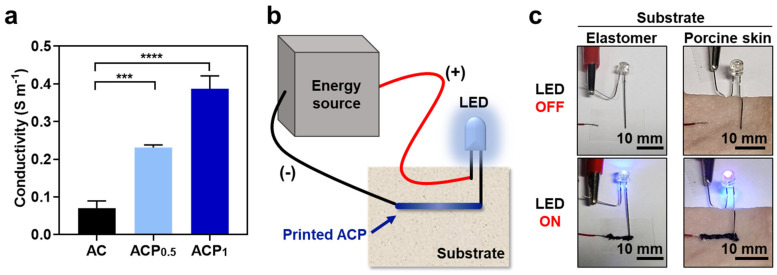
Electrical characterization of AC and ACPs. (**a**) Conductivity of AC (black), ACP_0.5_ (light blue), and ACP_1_ (dark blue). (**b**) Schematic of LED-emitting experiment on variable substrates. (**c**) LED emission in electrical circuit serially connected with ACP_1_ printed on elastomer substrate and porcine skin substrate. One-way ANOVA, *** p < 0.001, **** p < 0.0001.

Electrical characterization of the ACPs was also conducted to prove its versatility for future applications in bioelectronics. While the AC showed low conductivity (e.g., 0.07 S m^−1^), the conductivity of the ACP was at least three times as high (Figure 5a). As expected, ACP_1_, which had a higher PEDOT:PSS ratio, showed higher conductivity than ACP_0.5_. Therefore, we can choose either ACP_0.5_ or ACP_1_ as required. If a smooth filament with high injectability is more important than conductivity, we can use ACP_0.5_. Alternatively, if the conductivity is more important, we can use ACP_1_ because it can be injected while the filament is rough.

The conductivity of the ACP was further demonstrated using light-emitting diodes (LEDs). As illustrated in Figure 5b, one side of the LED was connected to the energy source, and the other side was affixed to the substrate without connection. Because catechol moieties can be attached to hydrophobic or hydrophilic substrates, we prepared a hydrophobic elastomer and porcine skin tissue as the experimental substrates. The disconnected side of the LED was connected to the energy source by printing the ACP_1_ to light the LED. The ACP effectively functioned as an electrode on both the elastomer and skin tissue surface (Figure 5c). These results suggest that the ACP can be used for flexible electronics, which can be further used for bioelectronics.

### 2.6. In Vitro Cell Viability of AC and ACPs

**Figure 6:**
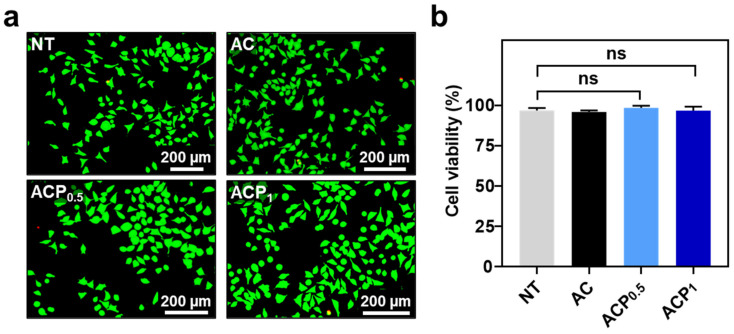
In vitro cytotoxicity of AC and ACPs. (**a**) Fluorescent images of L929 cells at 24 h after the treatment of the elutes (0.1×) from AC, ACP_0.5_, and ACP_1_. ‘NT’ as none of treatment. (**b**) Quantitative analysis of the cell viability (%). One-way ANOVA. ‘ns’ means ‘not significant’.

The ACPs can be potentially useful for versatile biomedical applications (e.g., bioelectronics). Thus, we evaluated in vitro cytotoxicity of the materials using mouse fibroblast cells (L929) (Figure 6). As expected, when the elutes from the hydrogels (AC, ACP_0.5_, and ACP_1_) were treated in the cells, approximately 97% of cells were alive for all groups (Figure 6b). The results correspond to those mentioned in the previous reports regarding Alg-CA and PEDOT:PSS, indicating great biocompatibility of the hydrogels [33].

## 3. Conclusions

A flexible PEDOT-based alginate granular hydrogel exhibiting tissue-adhesive properties was developed by employing a mussel-inspired catechol-functionalized material. The increase in the PEDOT concentration in the pre-gel solution changed the structure of the granular hydrogel from spherical to fiber-shaped with a higher aspect ratio. The hydrogel with a higher concentration of PEDOT (ACP_1_) exhibited a higher modulus and shear viscosity. These differences between ACP_0.5_ and ACP_1_ caused the morphology of the filament to differ: ACP_0.5_ formed a smooth filament when injected through a fine needle, whereas ACP_1_ formed a rough filament. The ACPs show greater tissue adhesiveness than the AC because the addition of PEDOT:PSS increases the viscosity of the hydrogel. The electrical conductivity of the ACPs was evaluated for further use as bioelectronic materials. ACP_1_ has a higher concentration of PEDOT, which leads to higher conductivity. The electrical performance was confirmed by illuminating a LED connected to an energy source using the ACP as the electrode. In addition, the biocompatibility of hydrogels was great for further application in the fields of flexible bioelectronics. Overall, the versatility of the ACP guarantees its use in various applications where conductive and soft materials are required.

## 4. Materials and Methods

### 4.1. Materials

1-(3-Dimethylaminopropyl)-3-ethylcarbodiimide hydrochloride (EDC-HCl) was purchased from Tokyo Chemical Industry (Tokyo, Japan). Bis(3-aminopropyl) terminated poly(dimethylsiloxane) (H2N-PDMS-NH2, Mn = 5000–7000) were purchased from Gelest All other chemicals were purchased from Sigma-Aldrich unless stated otherwise.

### 4.2. Synthesis of Catechol Conjugated Alginate (Alg-CA)

Alg-CA was prepared via an EDC/N-hydroxysulfosuccinimide (NHS) coupling reaction at pH 4.5–5. To synthesize Alg-CA, sodium alginate (Alg, from brown algae, medium viscosity, 1g) was dissolved in 2-Morpholinoethanesulfonic (MES) buffer solution (1.95%, pH adjusted to 4.6, 100 mL) at a concentration of 1% (*w*/*v*). The solution was purged with N_2_ for at least 10 min. The EDC (887 mg), NHS (532.5 mg), and dopamine hydrochloride (877.5 mg) were sequentially added to the Alg solution. The reaction proceeded for 8 h at room temperature. To remove the unreacted reagents, the solution was dialyzed against distilled water (pH adjusted to 4–5) for 24 h and lyophilized. The degree of conjugation was measured using a UV-vis spectrometer (Agilent 8453, Agilent Technology, Santa Clara, CA, USA) by constructing a standard curve using the absorbance values of the 10, 15, 25, 30, and 50 µg·mL^−1^ at 278 nm. The ^1^H NMR spectra of Alg and Alg-CA were obtained using Varian Oxford 300 Hz NMR (Varian, Palo alto, CA, USA). The polymer, Alg or Alg-CA, was uniformly dissolved in deuterium oxide (D_2_O) solution at the concentration of 10 mg·mL^−1^. The data was processed with MestReNova software (Mestrelab Research, Santiago de Compostela, A Coruña, Spain). Additionally, Fourier transform infrared (FT-IR) spectroscopy (Bruker IFS-66/S TENSOR 27 FT-IR spectrophotometer, Bruker Corp., Billerica, MA, USA) was utilized to analyze the catechol conjugation on the polymer backbone. The lyophilized samples were physically grinded and distributed in potassium bromide pellets for the FT-IR analysis. 

### 4.3. Fabrication of Alginate-Catechol Hydrogel (AC) and PEDOT:PSS Mixed Alginate-Catechol Granular Hydrogels (ACPs)

To fabricate the Alg bead, AC, and ACPs, a 2% (*w*/*v*) calcium chloride solution was prepared using deionized water. The lyophilized Alg-CA polymer (30 mg) and the PEDOT:PSS solution (1% stock solution) were used. A total of 0.5 mL of 1% PEDOT:PSS stock solution was mixed with 0.5 mL of deionized water to make 0.5% PEDOT:PSS solution. To fabricate ACP_0.5_ and ACP_1_, 30 mg of Alg-CA were dissolved in 0.5% (for ACP_0.5_) and 1% (for ACP_1_) PEDOT:PSS solution. To obtain the AC and ACPs, each of prepared solutions was dropped into a CaCl_2_ solution through a 26-gauge needle using a syringe pump (flow rate: 0.1 mL·min^−1^, NE-1000, New Era Pump Systems Inc., New York, NY, USA) and a DC high-voltage generator (NNC-HV30, Seoul, Republic of Korea); the distance between the needle tip and the collecting bath was 10 cm. 

### 4.4. Visualization of Granular Hydrogels

To optimize the hydrogel fabrication conditions, the voltages were varied as 5, 10, and 15 kV. An Alg-CA solution (3%) mixed with fluorescein isothiocyanate dextran (0.1%) was dropped into the CaCl_2_ solution. Fluorescence microscopy (DMi8, Leica, Wetzlar, Germany) was used for morphological imaging of the AC. The sizes and aspect ratios of the ACPs were measured using a Nikon Eclipse Ts2 optical microscope equipped with a Nikon DS-Fi3 microscope camera (Nikon, Tokyo, Japan) and ImageJ software (National Institutes of Health, Bethesda, MD, USA).

### 4.5. Morphological Analysis of AC and ACPs

For morphological analysis of the AC and ACPs, we utilized field emission scanning electron microscopy (FE-SEM, JSM-IT800, JEOL, Japan). The AC, ACP_0.5_, and ACP_1_ were lyophilized and cross-sectioned using a blade. The pore size in internal structure of hydrogels was measured at randomly chosen positions using ImageJ software.

### 4.6. Rheological Characterization of AC and ACPs

The rheological properties of the AC and ACPs were determined through oscillation frequency sweep tests, self-healing measurements, and step-strain test using Discovery Hybrid Rheometer 2 (TA Instruments, USA). All rheological measurements were conducted using a 20-mm parallel-plate geometry with a gap size of 300 µm. The storage (*G′*) and loss (*G″*) moduli were measured at room temperature under oscillation frequency sweeps (0.1–10 Hz, at 1% strain). The storage modulus and tan (δ) at 1 Hz were considered. To demonstrate the self-healing properties, G′ and G″ were measured under repeated application of 0.5 and 1000% strains for 180 s, respectively, at an osculation frequency of 1 Hz. The shear-thinning behavior of the ACP was explored by measuring the shear viscosity while continuously ramping the shear rate from 0 to 50 s^−1^. 

### 4.7. Tissue Adhesiveness of AC and ACPs

The tissue adhesion strengths of the AC and ACPs were investigated using a UTM (34SC-1, Instron, Norwood, MA, USA). For the shear stress test, 50 µL of each sample was loaded between two sides of porcine skin tissue (10 × 10 mm^2^). For the normal stress test, 200 µL of each sample was loaded between two sides of porcine skin tissue (20 × 20 mm^2^). The samples were stretched at a speed of 10 mm·min^−1^,and the adhesion strength (kPa) was calculated by dividing the maximum load (N) by the attached area (m^2^). 

### 4.8. Injectability and On-Tissue Printability of ACPs

The injectability of the ACPs was evaluated as follows: ACP_0.5_ and ACP_1_ were injected using 1 mL syringe equipped with a 23-gauge needle. To test the on-tissue printability of the ACP, ACP_0.5_ was used. Similar to the injectability test, the hydrogel was printed on porcine skin tissue using 1 mL syringe equipped with a 23-gauge blunt tip needle. The hydrogel-printed tissue was bent in both the concave and convex directions.

### 4.9. Electrical Characterization of AC and ACPs

The electrical properties of the AC and ACPs were measured using a digital multimeter (Keithley 2450 Digital Multimeter, Clackamas, OR, USA). The conductivity of the samples was measured via two-probe measurement and calculated using the following equation.

σ = L/(ρ × A)

where σ is the conductivity (S m^−1^), ρ is the resistance (Ω), A is the cross-section of the hydrogels (m^2^)), and L is the length of the hydrogels (m).

To demonstrate the conductive and injectable properties of ACP, ACP_1_ was injected on the elastomer synthesized as previously reported [35] using bis(3-aminopropyl) terminated poly(dimethylsiloxane) (H_2_N-PDMS-NH_2_, Mn = 5000–7000) or porcine skin using a 1 mL syringe equipped with a 23-gauge blunt needle tip to connect the LED and power supply (Keithley 2450 Digital Multimeter, Clackamas, OR, USA).

### 4.10. In Vitro Cytotoxicity Test

To evaluate in vitro cytocompatibility of the hydrogels, mouse fibroblast cells (L929) were pre-cultured in growth media (Dulbecco modified eagle medium (DMEM, low glucose, Gibco, USA) supplemented with 10% (*v*/*v*) fetal bovine serum (FBS, Gibco, USA) and 1% (*v*/*v*) penicillin–streptomycin (Gibco, USA)). The releasates from the AC, ACP_0.5_, and ACP_1_ (100 µL) were collected in DMEM (10 mL) for 24 h at 37 °C. The cells were seeded in a 48-well plate (1 × 10⁴ cells per well) and cultured overnight in a 5% humidified CO_2_ incubator at 37 °C. After washing with Dulbecco’s Phosphate-Buffered Saline (DPBS), the media containing the releasates, ten-fold diluted, was supplemented in each well. The cell viability was evaluated at 24 h using Live/Dead assay kit (Thermo Fisher Sci., Seoul, Republic of Korea). The cells were incubated in Calcein AM solution (2 μM) and Ethidium homodimer-1 solution (4 μM) (0.2 mL of total working solution) for 1 h at 37 °C. Finally, the live/dead cells were observed using fluorescence microscopy (DMi8, Leica, Wetzlar, Germany). The number of either green dots for live cells or red dots for dead cells was counted using ImageJ software, and cell viability (%) was calculated as the ratio of the number of live cells to the total number of cells.

### 4.11. Statistical Analysis

The statistical significance was evaluated using a one-way ANOVA with a post-hoc Tukey test. All data are expressed as the mean ± standard deviation. All experiments were conducted at least three times.

## Figures and Tables

**Table 1:** Experimental composition condition for fabricating AC and ACPs.

	Alg-CA (mg)	PEDOT:PSS 1% Solution (mL)	DDW (mL)
AC	30	0	1
ACP_0.5_	30	0.5	0.5
ACP_1_	30	1	0

## Data Availability

The data presented in this study are available in the article.

## Supplementary Materials

The following supporting information can be downloaded at: https://www.mdpi.com/article/10.3390/gels9020167/s1, Figure S1. (a) UV-Vis spectra of fabricated Alg-CA. The degree of conjugation of dopamine on alginate was calculated by UV absorbance value at 278 nm. (b) ^1^H NMR spectrum of Alg (black) and Alg-CA (red). The peaks at 6.5—7.1ppm indicate ‘a’ protons present in aromatic rings of catechol groups. c. FT-IR spectra of alginate (black) and Alg-CA (red). By conjugation of catechol on the polymer, the O-H stretching peak at 3406 cm^−1^ for Alg was shifted to 3366 cm^−1^ for Alg-CA, C-O stretching peak of carboxylic acid at 1414 cm^−1^ for Alg was shifted to 1408 cm^−1^ for Alg-CA, and O-H stretching peak of phenol appeared at 1290 cm^−1^ in Alg-CA spectra. Figure S2. (a-c). Self-healing property of hydrogels. Rheological measurement for self-healing property of AC (a), ACP_0.5_ (b), and ACP_1_ (c). Figure S3. Cross-sectional SEM analysis of AC and ACPs. (a) The SEM images of AC (left), ACP_0.5_ (middle), and ACP_1_ (right). (b) Quantification of the pore size of AC (black), ACP_0.5_ (light blue), and ACP_1_ (blue). One-way ANOVA, **** *p* < 0.0001.
