# Supplementary material for: Conductive and Adhesive Granular Alginate Hydrogels for On-Tissue Writable Bioelectronics
Source: Gels. 2023 Feb 19;9(2):167. doi: 10.3390/gels9020167 (PMC9957464; doi:10.3390/gels9020167)
Supplement: Supplementary file 1 [file gels-09-00167-s001.zip › gels-2220215-supplementary.pdf]

gels

Article

Conductive and Adhesive Granular Alginate Hydrogels for On-Tissue Writable Bioelectronics

Sumin Kim [1], Heewon Choi [2], Donghee Son [2,3,*] and Mikyung Shin [1,4,*]

[1] Department of Intelligent Precision Healthcare Convergence, Sungkyunkwan University (SKKU), Suwon 16419, Korea
[2] Department of Electrical and Computer Engineering, Sungkyunkwan University (SKKU), Suwon 16419, Korea
[3] Department of Superintelligence Engineering, Sungkyunkwan University (SKKU), Suwon 16419, Korea
[4] Department of Biomedical Engineering, Sungkyunkwan University (SKKU), Suwon 16419, Korea
* Correspondence: daniel3600@g.skku.edu (D.S.); mikyungshin@g.skku.edu (M.S.)

Supplementary Materials

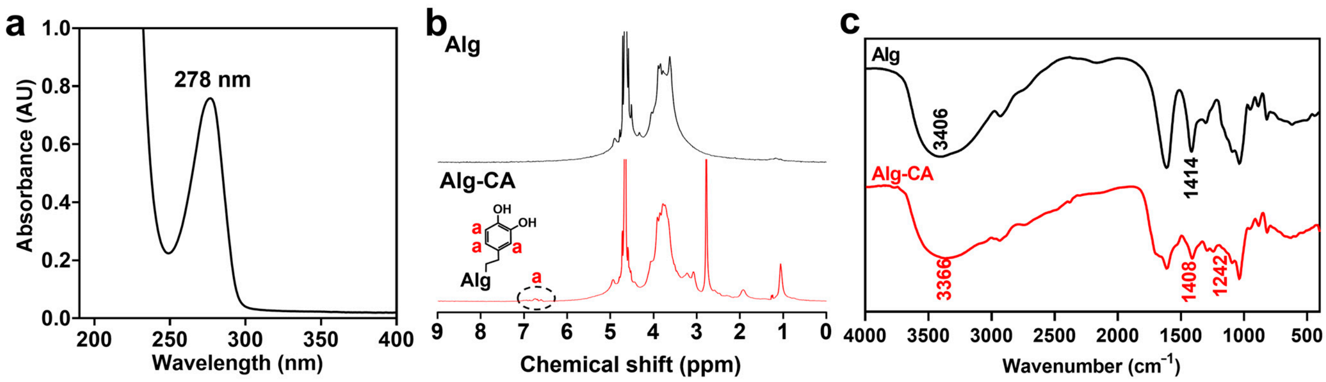

Figure S1. UV-Vis spectra of fabricated Alg-CA. **a**. The degree of conjugation of dopamine on alginate was calculated by UV absorbance value at 278 nm. **b**. ^{1}H NMR spectrum of Alg (black) and Alg-CA (red). The peaks at 6.5—7.1ppm indicate 'a' protons present in aromatic rings of catechol groups. **c**. FT-IR spectra of Alg (black) and Alg-CA (red). By conjugation of catechol on the polymer, the O-H stretching peak at 3406 cm^{-1} for Alg was shifted to 3366 cm^{-1} for Alg-CA, C-O stretching peak of carboxylic acid at 1414 cm^{-1} for Alg was shifted to 1408 cm^{-1} for Alg-CA, O-H stretching peak of phenol appeared at 1290 cm^{-1} in Alg-CA spectra.

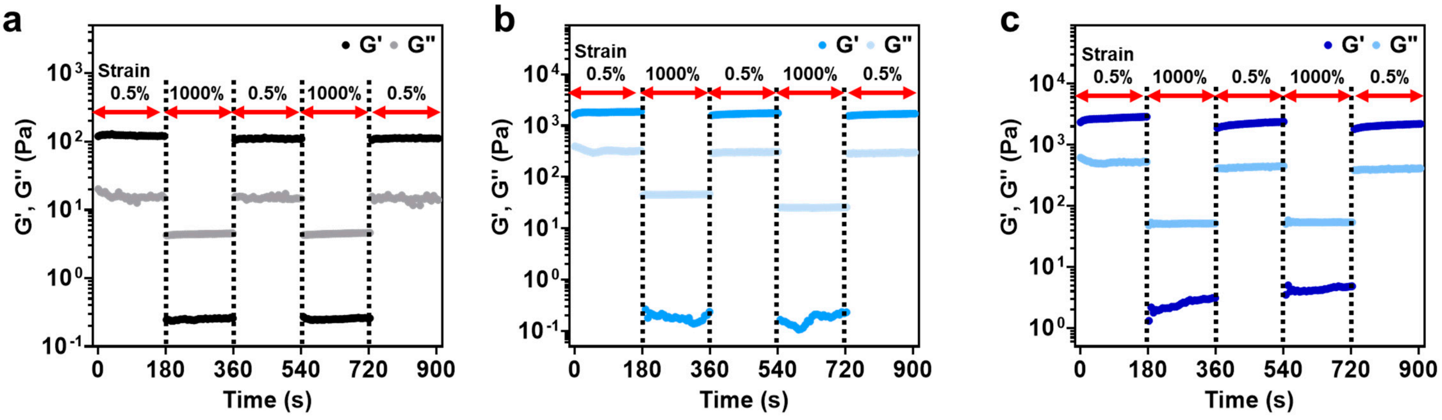

Figure S2. Self-healing property of hydrogels. a-c. Rheological measurement for self-healing property of AC (**a**), $ACP_{0.5}$ (**b**), and ACP_1 (**c**).

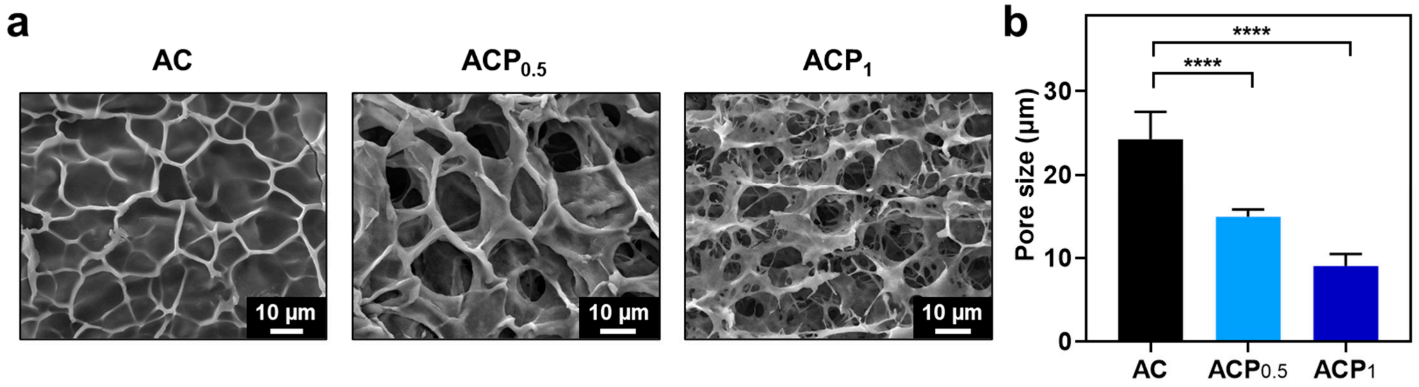

Figure S3. Cross-sectional SEM analysis of AC and ACPs. **a**. The SEM images of AC (left), $ACP_{0.5}$ (middle), and ACP_1 (right). **b**. Quantification of the pore size of AC (black), $ACP_{0.5}$ (light blue), and ACP_1 (blue). One-way ANOVA, ****$p < 0.0001$.
